# *P16*-specific DNA methylation by engineered zinc finger methyltransferase inactivates gene transcription and promotes cancer metastasis

**DOI:** 10.1186/s13059-015-0819-6

**Published:** 2015-11-23

**Authors:** Chenghua Cui, Ying Gan, Liankun Gu, James Wilson, Zhaojun Liu, Baozhen Zhang, Dajun Deng

**Affiliations:** Key Laboratory of Carcinogenesis and Translational Research (Ministry of Education/Beijing), Department of Aetiology, Peking University Cancer Hospital & Institute, Beijing, 100142 China; Department of Pathology, Institute of Hematology & Hospital of Blood Diseases, Chinese Academy of Medical Sciences, Tianjin, 300020 China; GRU Cancer Center, Georgia Regents University, Augusta, GA30912 USA

**Keywords:** Cancer, Engineered methyltransferase, Metastasis, Methylation, *P16*

## Abstract

**Background:**

*P16* DNA methylation is well known to be the most frequent event in cancer development. It has been reported that genetic inactivation of *P16* drives cancer growth and metastasis, however, whether *P16* DNA methylation is truly a driver in cancer metastasis remains unknown.

**Results:**

A *P16*-specific DNA methyltransferase (*P16-dnmt*) expression vector is designed using a *P16* promoter-specific engineered zinc finger protein fused with the catalytic domain of *dnmt3a*. *P16-dnmt* transfection significantly decreases *P16* promoter activity, induces complete methylation of *P16* CpG islands, and inactivates *P16* transcription in the HEK293T cell line. The P16-Dnmt coding fragment is integrated into an expression controllable vector and used to induce *P16*-specific DNA methylation in GES-1 and BGC823 cell lines. Transwell assays show enhanced migration and invasion of these cancer cells following *P16*-specific DNA methylation. Such effects are not observed in the *P16* mutant A549 cell line. These results are confirmed using an experimental mouse pneumonic metastasis model. Moreover, enforced overexpression of *P16* in these cells reverses the migration phenotype. Increased levels of RB phosphorylation and NFκB subunit *P65* expression are also seen following *P16*-specific methylation and might further contribute to cancer metastasis.

**Conclusion:**

*P16* methylation could directly inactivate gene transcription and drive cancer metastasis.

**Electronic supplementary material:**

The online version of this article (doi:10.1186/s13059-015-0819-6) contains supplementary material, which is available to authorized users.

## Background

*P16* (*CDKN2A* or *Ink4a*) is one of the most frequently deleted genes in cancer genomes and has been studied extensively [1]. *P16* germline mutation carriers have been shown to have a greatly increased predisposition to familial melanoma [2–4]. Recently, genetic inactivation of *P16* has been proven to be a driver for cancer metastasis in mice [5].

While genetic alterations in *P16* do occur, gene methylation is far more prevalent in human cancers [6–10]. Studies have shown that *P16* DNA methylation is correlated with a decreased level of expression in tissues [6–10] and is linked to the development and metastasis of many cancers [11–15]. It is therefore highly likely that *P16* DNA methylation may play an important role in cancer development.

It has been reported that artificial *P16* DNA methylation induced through the insertion of *alu* motifs increased the susceptibility of mice to developing cancer [16]. However, whether *P16* DNA methylation drives cancer metastasis has not been characterized. In the present study, a *P16*-specific DNA methyltransferase (*P16-dnmt*) was used to directly inactivate *P16* transcription and the subsequent effects on proliferation, migration, and invasion of cancer cells were evaluated *in vitro*. These results were further confirmed in immuno-deficient mice. This study provides experimental evidence that strongly implicates *P16* DNA methylation as a driver in cancer metastasis.

## Results

### *P16* DNA methylation directly inactivates gene transcription

**Fig. 1:**
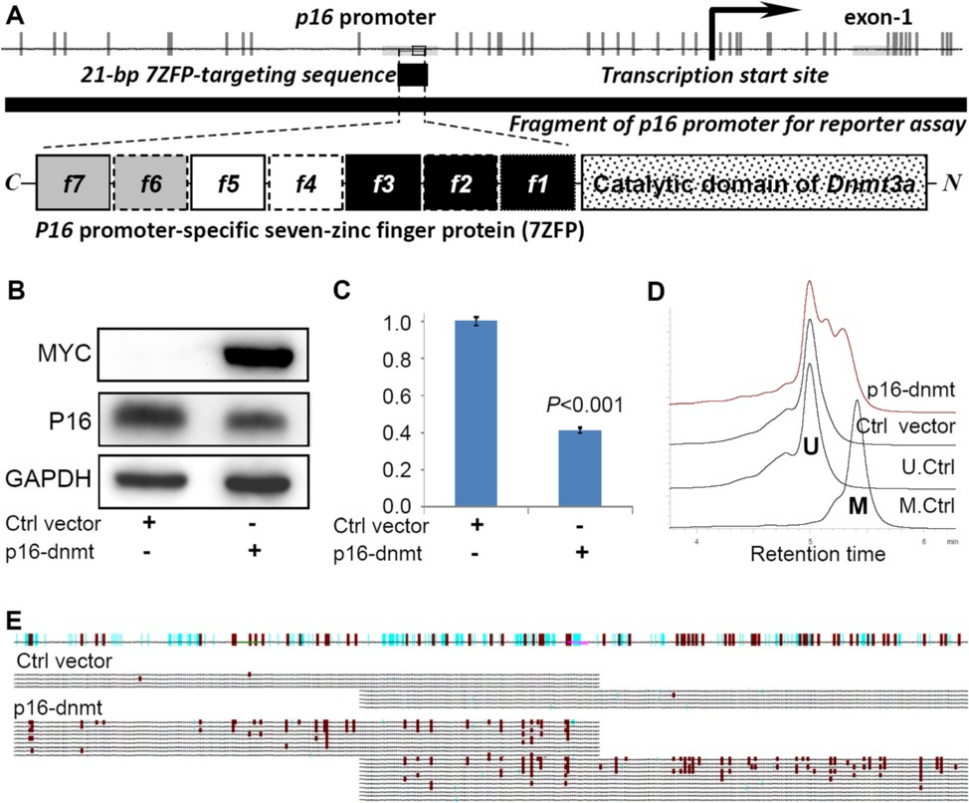
*P16*-specific methyltransferase (*P16-dnmt*) induces methylation of *P16* CpG islands and represses gene transcription in HEK293T cells. **a** Construction of *P16-dnmt* using *P16* promoter*-*specific seven-zinc finger protein (7ZFP) and the catalytic domain of mouse *DNMT3a*; **b** Western blot analysis for P16-Dnmt and endogenous P16; **c** Reporter assay results following *P16-dnmt* transfection; **d** DHPLC methylation analysis of the *P16* promoter in HEK293T cells; the 392-bp methylated (M) and unmethylated (U) *P16* fragments were detected at the partial denaturing temperature of 57.0 °C; **e** bisulfite clone-sequencing results from HEK293T cells transiently transfected with *P16-dnmt*

**Fig. 2:**
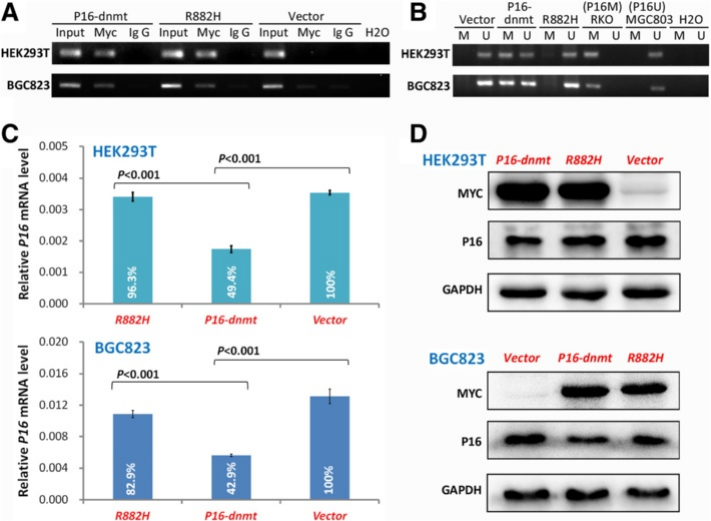
Comparison of DNA binding capacity, DNA methylation induction, and *P16* expression levels between *P16-dnmt* and the R882H mutant in HEK293T and BGC823 cells 72 h following transient transfection. **a** Chromatin-immunoprecipitation (ChIP)-PCR results comparing *P16* promoter DNA binding for P16-Dnmt and R882H protein; **b** Methylation-specific PCR (MSP) detecting methylated and unmethylated *P16* alleles; **c** Quantitative RT-PCR indicating *P16* mRNA levels; **d** Western blot of P16 and Myc/P16-Dnmt protein levels

In order to determine whether *P16* DNA methylation directly inactivates gene transcription, a *P16* promoter-specific DNA methyltransferase (*P16-dnmt*) was initially constructed using the pcDNA3.1_myc/His vector as described in the methods section (Fig. 1a). Western blot analysis confirmed that endogenous P16 was greatly reduced in HEK293T cells 48 h following transient transfection with the *P16-dnmt* vector (Fig. 1b). A dual-luciferase reporter assay further illustrated that *P16* promoter activity was significantly inhibited in the *P16-dnmt-*transfected cells (Fig. 1c). Notably, methylation of CpG islands within both the *P16* promoter and exon-1 regions was detected using denatured high performance liquid chromatography (DHPLC) and bisulfite-sequencing (Fig. 1d and **e**). An additional control lacking approximately 80 % of the DNA methyltransferase activity (R882H mutant) was constructed to evaluate the impact of steric hindrance from P16-Dnmt DNA binding on gene transcription. As expected, chromatin-immunoprecipitation (ChIP)-PCR analysis showed that the mutant still bound with the *P16* promoter DNA fragment (Fig. 2a), but did not induce *P16* DNA methylation (Fig. 2b). Furthermore, its capacity to repress *P16* expression was sharply decreased in both HEK293T and BGC823 cells (Fig. 2c and d). These data suggest that *P16* DNA methylation is directly responsible for *P16* repression as opposed to steric hindrance. Taken together, these results indicate that *P16-dnmt* encodes an active methyltransferase for *P16* CpG islands, and *P16* DNA methylation is sufficient to inactivate endogenous *P16* expression.

**Fig. 3:**
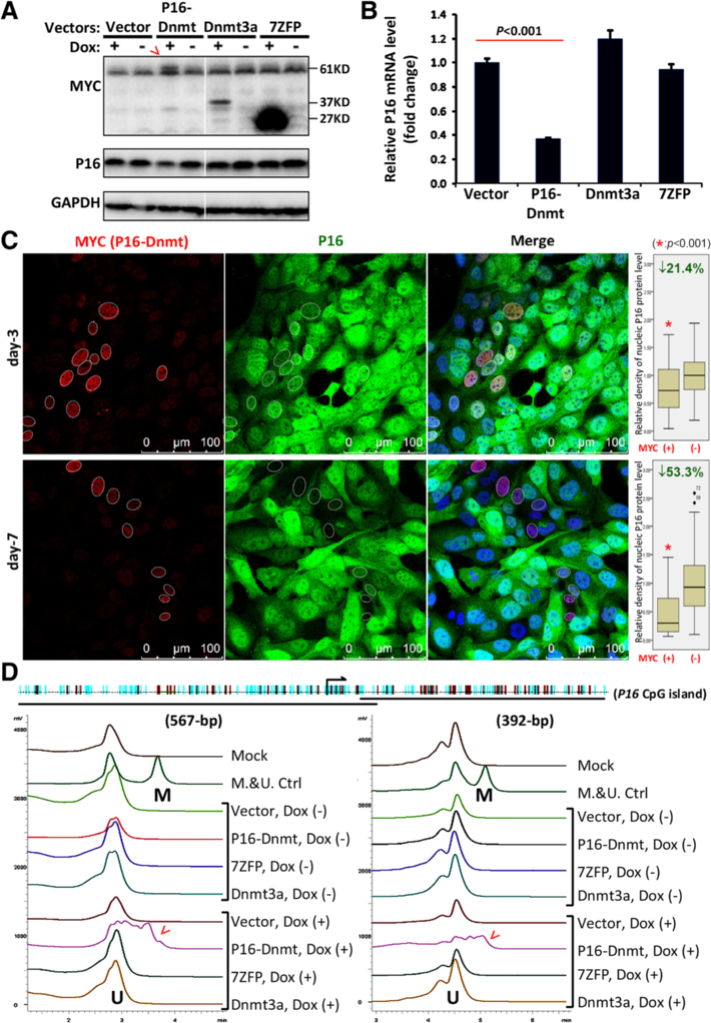
*P16* DNA methylation analysis of GES-1 cells stably transfected with the *P16-dnmt* pTRIPZ ‘Tet-on’ vector. **a** Western blot analysis of P16 and P16-Dnmt (61KD, arrow) and control vectors following treatment with 0.25 μg/mL doxycycline for 3 days. **b** Quantitative RT-PCR results for the *P16-dnmt* stably transfected, doxycycline-treated cells. **c** Confocal analysis with P16 labeling following 3 and 7 days of doxycycline treatment. **d** Confirmatory DHPLC methylation analysis of *P16*-specific methyltransferase expressing cells (arrow) and controls following 7 days of doxycycline treatment. The 567-bp methylated (M) and unmethylated (U) *P16* promoter fragments were analyzed at the partial denaturing temperature of 54.0 °C. DNA samples from HCT116 cells containing both the methylated and unmethylated *P16* alleles were used as controls (M.&U.Ctrl)

In order to specifically methylate *P16* CpG islands, the *P16-dnmt* coding-sequence was then integrated into the pTRIPZ lentivirus vector carrying a ‘Tet-on’ switch to allow the gene expression to be controlled. Expression of P16-Dnmt protein was induced in GES-1 cells stably transfected with the *P16-dnmt* pTRIPZ vector after treatment with 0.25 μg/mL doxycycline for 3 days (61KD; Fig. 3a). Significant inhibition of endogenous *P16* expression was observed in Western blot and quantitative RT-PCR analysis when compared to GES-1 cells transfected with the *dnmt3a* and *7ZFP* control vectors (Fig. 3a and b). Confocal microscopy revealed that the average density of nucleic P16 gradually decreased in the *P16-dnmt* expressing cells (Fig. 3c). In fact, after treatment with doxycycline for 3 and 7 days, *P16* expression levels were decreased by 21.4 % and 53.3 %, respectively (*P* <0.001). Most importantly, intensive methylation of *P16* CpG islands was induced in the GES-1 cells stably transfected with *P16-dnmt* and treated with doxycycline, but not in cells transfected with the control vectors, nor in cells that did not receive doxycycline treatment (Fig. 3d). Similarly, *P16* DNA methylation and subsequent repression of *P16* expression was also induced by P16-Dnmt in the BGC823 cell line (Additional file 1: Figure S1).

**Fig. 4:**
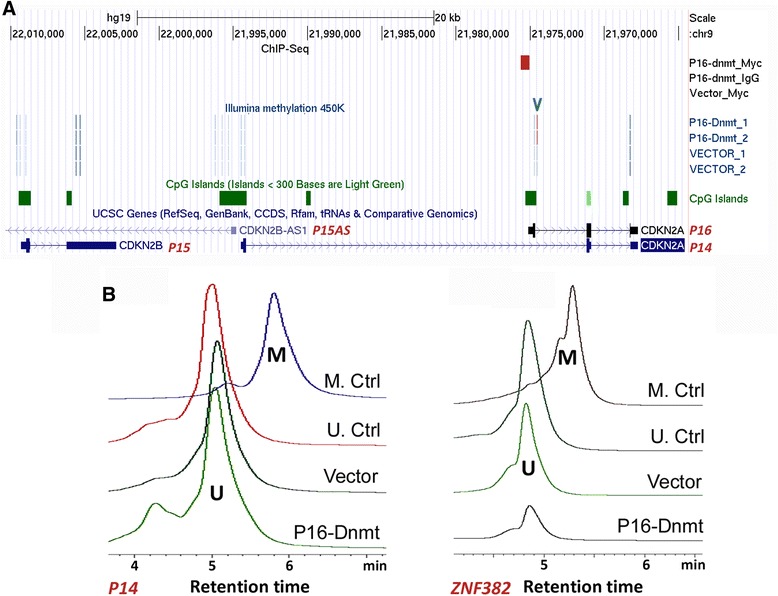
Characterization of the P16-Dnmt binding fragments and methylation status of the promoter CpG islands in *P16* and control genes in *P16-dnmt* stably transfected cells. **a** Chromatin-immunoprecipitation-sequencing (ChIP-Seq) and Illumina methylation 450 K array results in the *ink4a* locus in BGC823 cells; **b** DHPLC methylation analysis of CpG islands in *P14* and *ZNF382* promoters in GES-1 cells expressing P16-Dnmt. DNA samples with and without *M.sss*I methylation were used as methylated and unmethylated controls

ChIP-PCR analysis also showed that P16-Dnmt specifically bound the *P16* promoter, but not the *P14* promoter (Additional file 1: Figure S2). Similarly, ChIP-sequencing confirmed that the P16-Dnmt binding fragment was only detected in the promoter of P16-Dnmt/Myc antibody immunoprecipitated DNA from the *P16-dnmt*-expressing BGC823 cells, but not in the IgG control, nor the cells transfected with the control vector (Fig. 4a, red fragment; Additional file 2: File S1, Additional file 3: File S2, and Additional file 4: File S3). Although most of the P16-Dnmt binding fragments were found in intergenic and intron sequences (Additional file 1: Figure S3A), the main P16-Dnmt binding motif was found to closely match the antisense strand of the targeted fragment in the *P16* promoter with a similarity of 21/23 (91.3 %) base pairs (Additional file 1: Figure S3C, red-framed motif). Genome-wide methylation analysis of P16-Dnmt expressing BGC823 cells was performed using an Infinium Methylation 450 K array. The results illustrated that 647 of 481,615 informative CpG sites (0.13 %) were significantly hypermethylated (Δβ >0.50). Interestingly, 229 of these 647 CpG sites were located in intragenic CpG islands and shores corresponding to 203 genes (Additional file 5: File S4). The targeted *P16* CpG island was included in the list of differentially hypermethylated sites (Fig. 4a, blue arrow). Furthermore, DNA methylation was not induced in the CpG islands of two control genes, *P14* (located within the same *CDKN2A* locus as *P16*) and *ZNF382* (located on a different chromosome) (Fig. 4b). These results suggest that doxycycline-induced *P16-dnmt* expression could specifically methylate *P16* CpG islands.

### *P16*-specific DNA methylation promotes migration and invasion of cancer cells

**Fig. 5:**
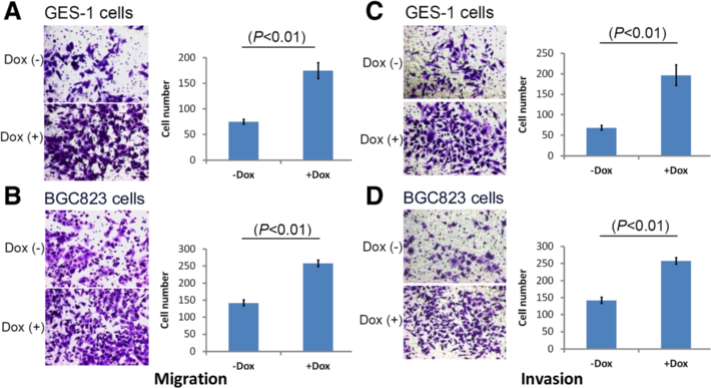
Migration and invasion assays carried out with cell lines stably transfected with the *P16-dnmt* pTRIPZ vector *in vitro*. **a**, **b** Results of Transwell migration assays for GES-1 and BGC823 cell lines following 48-h and 36-h incubation, respectively; **c**, **d** Results of Matrigel invasion assays for GES-1 and BGC823 cell lines following 108-h and 96-h incubation, respectively; the average cell number and s.d. are displayed (Right). Dox (+), with 0.25 μg/mL doxycycline treatment; Dox (−), without doxycycline treatment. These experiments were independently repeated in triplicate

Various assays were then conducted to further characterize the biological behaviors of cancer cells following *P16*-specific inactivation by DNA methylation. Transwell assays revealed that the migration ability of GES-1 and BGC823 cells was significantly increased following *P16*-specific DNA methylation (Fig. 5a and b). Similarly, Matrigel assays showed that the invasion capacity of these cell lines was also significantly enhanced by *P16-*specific DNA methylation (Fig. 5c and d).

**Fig. 6:**
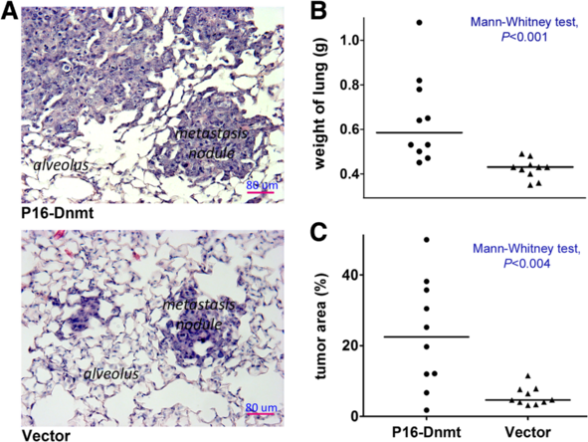
*P16*-specific methylation promotes experimental pneumonic metastasis of BGC823 cells. **a** Images of representative metastatic nodules in the lung of SCID mice (H&E staining). **b** The lung weights of mice in the *P16-dnmt* pTRIPZ and control groups at day 19. **c** The ratio of metastatic nodule area to lung area of mice in the *P16-dnmt* pTRIPZ and control groups

Four weeks after BGC823 cells stably transfected with *P16-dnmt* were injected into the tail vein of the NOD SCID mice, metastatic nodules were observed in the lung (Fig. 6a). The average lung weight, which correlates with the number of metastatic cells, in the *P16-dnmt* group was 152.5 % that of the empty vector control group (Mann–Whitney test, *P* <0.001; Fig. 6b). The average proportion of metastatic nodule area to total lung area in the *P16-dnmt* group was also significantly higher than the control group (*P* <0.004, Fig. 6c).

In addition, *P16*-specific DNA methylation was found to slightly, but significantly, inhibit proliferation of GES-1 cells, while the proliferation of BGC823 cells was not affected (Additional file 1: Figure S4). However, growth inhibition of the *P16-dnmt* transfected GES-1 cells was not observed in the SCID mice despite detection of methylated-*P16* alleles in the xenografts (Additional file 1: Figure S5).

**Fig. 7:**
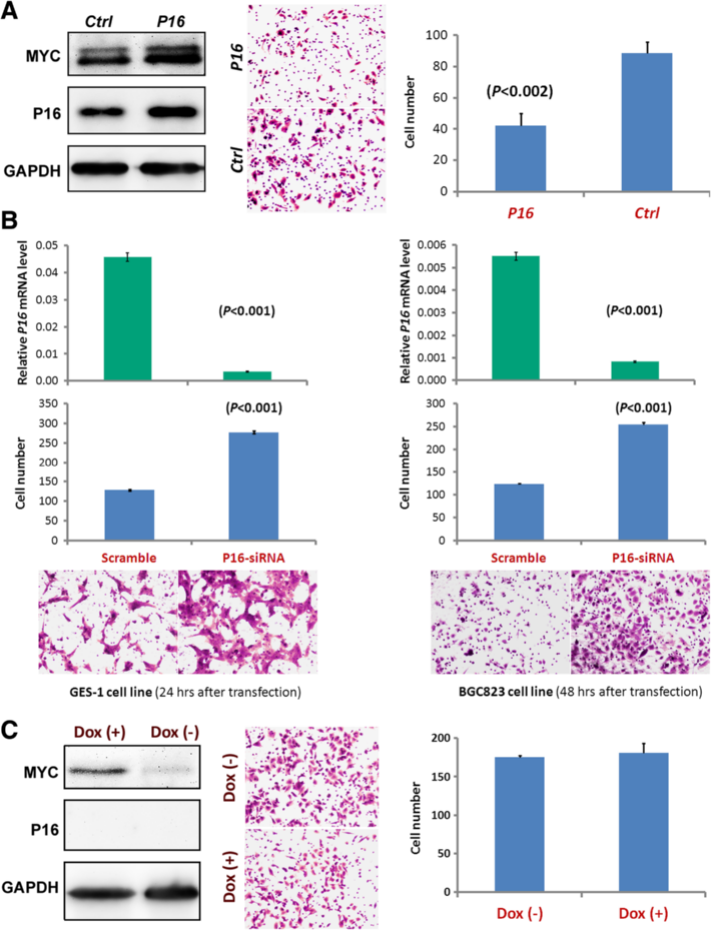
Effect of varying *P16* expression on migration. **a** Rescue assay demonstrating the effect of enforced *P16* overexpression on migration of the *P16-dnmt* stably transfected BGC823 cells treated with doxycycline for 2 weeks. **b** Migration assay results after 24-h and 48-h transient siRNA transfection in GES-1 and BGC823 cell lines. *P16* qRT-PCR results are also presented. **c** Migration capacity of A549 cells (lacking *P16* alleles) following stable transfection of *P16-dnmt*. After treatment with 0.25 μg/mL doxycycline for 1 week, these cells (4.0 × 10^4^) were seeded into each well and incubated for 28 h. Western blot analysis of P16-Dnmt and P16 expression is also presented. Migration assays were independently repeated in triplicate

In order to confirm whether the enhanced migration of cancer cells is *P16* DNA methylation-specific, a rescue assay was carried out in the *P16-dnmt* expressing BGC823 cells through transient transfection of a *P16* expression vector. Results of the Transwell assay demonstrated that enforced *P16* overexpression significantly reversed the enhanced migration phenotype of these cells (Fig. 7a). Similar results were also observed in HONE-1 cells (Additional file 1: Figure S6). In contrast, downregulation of endogenous *P16* expression through transient siRNA transfection significantly enhanced the migration of BGC823 and GES-1 cells (Fig. 7b). Furthermore, the migration capacity of A549 cells, which lack *P16* alleles, was not changed following stable transfection of *P16-dnmt* and 7 days of doxycycline treatment (Fig. 7c). Taken together, these results imply that the enhanced migration and invasion phenotypes of cancer cells are *P16*-specific.

### *P16*-specific DNA methylation promotes RB phosphorylation and upregulates NFκB subunit P65 expression

**Fig. 8:**
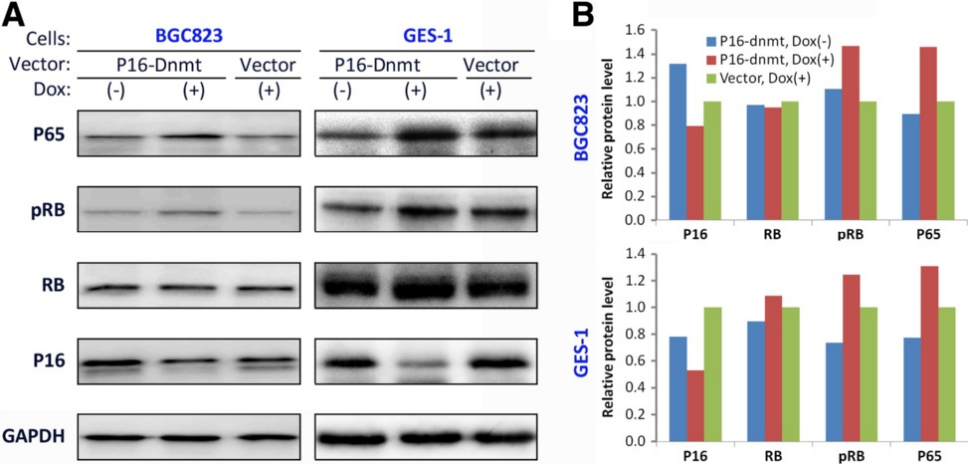
Western blot analysis of P16, RB, phosphorylated RB, and NFκB subunit P65 expression in the *P16-dnmt* stably transfected cells. BGC823 and GES-1 cell lines were tested 14 and 8 days following doxycycline treatment, respectively. **a** WB image; **b** Relative density of tested protein bands normalized against GAPDH

To confirm that *P16* DNA methylation affects its downstream signal pathway, P16-CDK4/6-RB, the phosphorylation level of RB protein was analyzed using Western blot analysis. As expected, increased levels of phosphorylated RB were detected in the *P16-dnmt* transfected BGC823 and GES-1 cells treated with doxycycline when compared to those without doxycycline induction and those transfected with the control vector. Total RB protein levels were not changed (Fig. 8a and b). Furthermore, the expression level of nuclear factor NFκB subunit P65 was also increased in the *P16-dnmt* transfected cells.

## Discussion

It is well known that methylation of CpG islands around transcription start sites is inversely correlated with the expression level of genes in many cells and tissues. *P16* DNA methylation may occur as a long-term mechanism to maintain gene suppression following transcriptional silence induced by repressive histone modifications [17]. Whether *P16* promoter methylation alone is capable of silencing transcription has not been well studied. In the present study, we found that P16-Dnmt-induced methylation of *P16* CpG islands could directly inactivate gene expression and promote metastasis of cancer cells.

Studies have shown that target-specific methylation/ demethylation is associated with transcriptional inactivation/re-activation of several other human genes [18–20]. Zhang *et al.* reported that *P16*-specific artificial transcription factor (P16-ATF) could induce *P16* DNA demethylation and re-activate its expression [21]; however, it is not known whether DNA demethylation is essential for re-activation of this gene. Yu *et al.* successfully established a *p16* DNA methylation model in mice through the insertion of *alu* motifs into the mouse *p16* promoter and found subsequent gene inactivation [16]; however, the possibility that the insertion of *alu* motifs directly contributed to transcriptional repression cannot be excluded*.* In order to increase the targeting specificity in the present study, we employed the pTRIPZ vector that allowed for controllable expression of P16-Dnmt. Our results showed that P16-Dnmt-induced DNA methylation was sufficient to silence transcription in two human gastric epithelial cell lines. This result is consistent with a recent report which demonstrated that engineered transcription activator-like effector (TALE)–Dnmts induced *P16* DNA methylation, inactivated gene expression, and increased replication in human fibroblasts [22]. Additionally, steric hindrance from P16-Dnmt DNA binding does not appear to play a significant role in repressing gene transcription as was demonstrated using the *P16-dnmt* R882H mutant control. Taken together, the evidence suggests that *P16* DNA methylation primarily accounts for the inactivation of *P16* transcription.

Genome-wide CRISPR screens in mouse models have shown that genetic *p16* inactivation may be a driver for tumor growth and metastasis [5]. Luo *et al*. have reported that the proportion of methylated *P16* alleles is significantly associated with metastasis of gastric cancers [13]. Zhang *et al.* have also suggested that re-activation of methylated *P16* by P16-ATF inhibits migration and invasion in AGS and H1299 cancer cell lines [21]. Here, we have provided evidence to demonstrate that P16-Dnmt mediated DNA methylation might promote metastasis of cancer cells *in vitro* and *in vivo*. Moreover, we found that such an effect was not observed in A549 cells lacking *P16* alleles, and siRNA downregulation of *P16* expression also promoted the migration of cell lines, and overexpression of *P16* reversed the cell migration phenotype. These facts strongly implicate inactivation of *P16* by DNA methylation as a possible promoter of migration/invasion and metastasis of cancer cells.

Inactivation of the *P16* gene results in higher cyclin D-dependent protein kinase activity and thus induces aberrant phosphorylation of RB protein. Therefore normal cell cycle checkpoints are bypassed allowing accelerated cell growth and increased genomic instability [23, 24]. We found that the induction of *P16*-specific DNA methylation could also increase the phosphorylation of RB.

NFκB subunit P65 is the master regulator in the senescence-associated secretary phenotype (SASP) [25]. In melanomas, the expression of P65 is increased while *P16* expression is decreased [26]. P65 also regulates the transcription of a group of metastasis-related genes, including MMP-9/2 [27–29]. In this study, we also found that induction of *P16* DNA methylation also increases the amount of P65 protein in cancer cells. Additional studies are required to determine other pathways involved in the *P16* DNA methylation-related metastasis phenotype.

## Conclusion

Engineered zinc finger protein-targeted *P16* DNA methylation directly inactivates *P16* expression and promotes invasion and metastasis of cancer cells.

## Methods

### Cell lines and cultures

HEK293T, BGC823, and GES-1 cell lines were kindly provided by Professor Yang Ke at Peking University Cancer Hospital and Institute. The A549 cell line lacking the *P16* locus was kindly provided by Professor Zhiqian Zhang at the same institute. The HONE-1 cell line was kindly provided by Professor Zhen Sun at Capital Medical University School of Stomatology, Beijing. All of these cell lines were tested and authenticated using the Goldeneye20A STR Identifiler PCR Amplification Kit (Beijing Jianlian Genes Technology Co., Ltd.) before being used in this study [30]. These cell lines were cultured in RPMI1640 medium supplemented with 10 % FBS and maintained at 37 °C in humidified air with 5 % CO_2_. Cell proliferation was analyzed using the Cell Counting Kit-8 (CCK-8) [21].

### Construction of vectors and transfection

The *P16-dnmt* plasmid was constructed by fusing a SP1-like engineered seven-zinc finger protein (7ZFP) *6I* capable of specifically binding the 21-bp fragment (5′-GAG GAA GGA AAC *GGG GCG GGG-3*′, including a *Sp1-binding site*) within the human *P16* promoter [21] with the catalytic domain (approximately 608–908aa) of mouse *dnmt3a* in the pFast Bac HT A-dnmt3a vector (kindly provided by Professor Keith Robertson at Georgia Regents University, USA) [31]. Point mutation R882H in the catalytic domain of *Dnmt3a* is the most frequent somatic mutation in acute myeloid leukemia [32]. The methyltransferase activity of R882H DNMT3A is reduced by approximately 80 % compared with the wide-type [33]. Thus, a *P16-dnmt* R882H mutant control was constructed as a negative control. The *P16-dnmt* coding sequence was integrated into a pcDNA3.1 vector and an expression controllable pTRIPZ vector carrying a ‘Tet-on’ switch (Open Biosystem, USA), respectively. Control vectors for the Dnmt3a catalytic domain or 7ZFP(6I) were also constructed. The purified *P16-dnmt* plasmid DNA was mixed with VSVG and Δ8.9 (Addgene, USA) to prepare lentivirus transfection particles. The *P16* expression vector was constructed using wild-type P16 coding sequence cDNA and integrated into the pIRES2-EGFP vector. The cells (4.5 × 10^4^) were transiently transfected with the pIRES2-*P16* expression vector, seeded into each well, and incubated for 43 h. *P16* specific siRNAs (5′-CCGUA AAUGU CCAUU UAUAT T-3′ and 5′-UAUAA AUGGA CAUUU ACGGT T-3′) were synthesized (GenePharma) and used to transiently transfect cells at a final concentration of 1.0 μg/1 mL. The scramble siRNAs (5′-UUCUC CGAAC GUGUC ACGUT T-3′ and 5′-ACGUG ACACG UUCGG AGAAT T-3′) were used as negative control. The fresh lentivirus particles were used to transfect human cells.

### Bisulfite-DHPLC, −sequencing, MethyLight, and methylation-specific PCR (MSP)

The 392-bp fragments isolated from the antisense-strand of *P16* exon-1 in cultured cells were amplified with a CpG-free primer set and analyzed using DHPLC and clone sequencing as described previously [13, 34]; however, the PCR annealing temperature was fixed at 57.0 °C to avoid amplification bias between methylated and unmethylated *P16* alleles. The 567-bp fragment in the antisense-strand of the *P16* promoter was also amplified using a CpG-free primer set (forward, 5′-gaatt agggt ttttg attta gtgaa tt-3′; reverse, 5′-accct atccc tcaaa tcctc taaa-3′) at an annealing temperature of 65 °C, analyzed at the partial denaturing temperature of 54 °C in DHPLC analysis, and confirmed using clone sequencing. Methylated and unmethylated *P16* were also analyzed by 150/151-bp MSP [35].

The 272-bp *P14* CpG island fragment was amplified using a CpG-free primer set (forward, 5′-gttgt ttatt tttgg tgtta-3′; reverse, 5′-acctt tccta cctaa tcttc-3′) at the annealing temperature of 51.0 °C, and analyzed at the partial denaturing temperature of 57.7 °C in DHPLC analysis. The 437-bp *ZNF382* CpG island fragment was amplified and analyzed by DHPLC as previously described [30].

### Quantitative RT-PCR, Western blot, and confocal analysis of *P16* expression

The *P16* mRNA and protein level in cell lines were analyzed as described [21].

### Chromatin immunoprecipitation (ChIP) assays

The 124-bp *P16* and 61-bp *P14* DNA fragments within CpG islands bound to P16-Dnmt were quantitated as described [21, 36]. Anti-Myc antibody was used to precipitate P16-Dnmt protein containing a Myc tag. The Myc-ChIPed-DNA samples were sequenced using the Illumina HiSeq2500 (Shanghai Biotechnology Co., China). The readouts were preprocessed using online fastx software (version 0.0.13; http://hannonlab.schl.edu/fastx_toolkit/index.html), mapped to the human genome hg19 using Bowties (version o.12.8) [37], and enriched using MACS (version 1.4.2) [38]. The protein-binding motif was identified using MEME software [39]. The detected peaks/annotated information is presented as Additional file 2: File S1, Additional file 3: File S2, and Additional file 4: File S3.

### Genome-wide analysis of DNA methylation

Illumina Infinium HD Methylation450K arrays were used to perform differential CpG methylation analyses on BGC823 cells stably transfected with the *P16-dnmt* and pTRIPZ control vectors following 14 days of doxycycline treatment according to the Assay Manual. Two parallel samples were tested for each group. DNA methylation levels for each CpG site were computed as the ratio of normalized methylated signal intensity to the sum of methylated and unmethylated signal intensities using GenomeStudio software. Using the control vector as a reference, Δβ was calculated to represent differential methylation for each CpG site in the *P16-dnmt *expressing cells. The differential methylation was considered to be significant when the Δβ value was >0.50. The raw methylation data are available as Additional file 6: File S5.

### Dual-luciferase reporter assay

The *P16* promoter (approximately −597 to +155 nt) was integrated into the pGL3-Basic vector and used for promoter activity analysis as previously described [21].

### Transwell migration and Matrigel invasion tests

The Transwell migration and Matrigel invasion tests were performed using GES-1 and BGC823 cells suspended in 150 μL serum-free medium (2 × 10^5^ cells/mL). The BGC823 cells were incubated for 36 h and 96 h at 37 °C in 5 % CO_2_ before quantifying their migration and invasion capacity, respectively. Similarly, the GES-1 cells were quantified following 48-h and 108-h incubation, respectively [21]. Wound healing status was dynamically recorded using the IncuCyte ZOOM™ live-cell imaging platform. Each trial consisted of three independent samples, and all the assays were repeated two to three times.

### Xenografts and pneumonic metastases in SCID mice

GES-1 cells (1.4 × 10^6^ cells in 200 μL Matrigel suspension) were stably transfected with the *P16-Dnmt* or control vector, induced with 0.25 μg/mL doxycycline for 7 days, and then injected subcutaneously into the lower limb of NOD SCID mice (female, 5 weeks old, weight 10–20 g, purchased from Beijing Huafukang Biotech). The mice were provided distilled, sterile water containing 2 μg/mL doxycycline. These mice were sacrificed 48 days after transplantation. The weight and volume of tumors were then analyzed.

For the pneumonic metastasis assay, BGC823 cells stably transfected with the *P16-dnmt* or control vector were also induced with 0.25 μg/mL doxycycline for 7 days, and then injected into the tail vein of the SCID mice (1.5 × 10^6^ cells in 0.15 mL medium) (10 randomly allocated mice per group). The lung weight was detected at the 19th experimental day for each mouse [40]. The lung organs were fixed with Bouin solution, paraffin-embedded and cut into 5 μm slides along the maximum area, and examined microscopically following H&E staining. The lung area and total tumor nodule area were measured using INFINITY Analyze (Version 4.0, Lumenera Sci). The nodule area to the lung area ratio was calculated for each mouse.

### Statistical analysis

Results were displayed by constituent ratios of enumeration or ranked data. All *P* values were two-sided, and a difference with *P* <0.05 was considered statistically significant.

### Ethical approval

This study was approved by the institute’s animal ethics committee (approval no. AE-2012-06).

### Data and materials availability

The methylation array data have been deposited into the Gene Expression Omnibus under accession number GSE74233. The ChIP-sequencing raw data have been deposited into the bioproject database under accession number SRA306603.

## Additional files

Additional file 1:
**Figures S1 to S6.** (DOC 3228 kb) Additional file 2:
**File S1 (annotated_tss_P16Dnmt-Myc.fq_macs_peaksFile).** (XLSX 1943 kb) Additional file 3:
**File S2 (annotated_tss_P16Dnmt-IgG.fq_macs_peaks).** (XLSX 139 kb) Additional file 4:
**File S3 (annotated_tss_vector-Myc.fq_macs_peaks).** (XLSX 153 kb) Additional file 5:
**File S4 (P16-Dnmt induced methylation sites).** (XLSX 4152 kb) Additional file 6:
**File S5 (450 K RawSignal).** (CSV 46821 kb)

## Abbreviations

7ZFP: seven zinc finger protein
DHPLC: denatured high performance liquid chromatography
Dox: doxycycline
P16-ATF: *P16*-specific transcription factor
P16-Dnmt: *P16*-specific methyltransferase
