# Supplementary material for: P16-specific DNA methylation by engineered zinc finger methyltransferase inactivates gene transcription and promotes cancer metastasis
Source: Genome Biol. 2015 Nov 23;16:252. doi: 10.1186/s13059-015-0819-6 (PMC4656189; doi:10.1186/s13059-015-0819-6)
Supplement: Additional file 1: — Figures S1 to S6. (DOC 3228 kb) [file 13059_2015_819_MOESM1_ESM.doc]

**
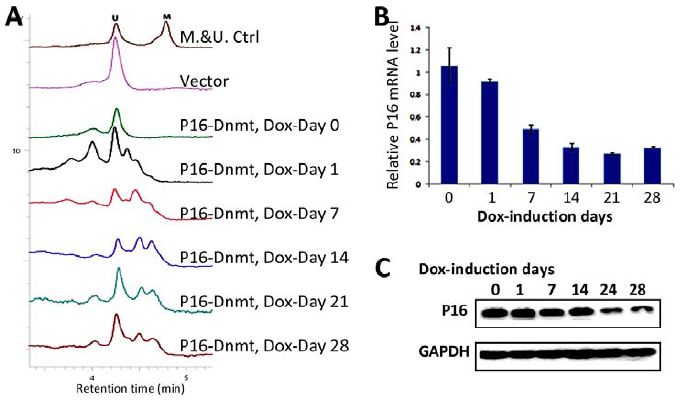
**

**Figure S1.** Dynamic alterations of *P16* DNA methylation, transcription, and protein expression in BGC823 cells stably transfected with the *P16*-*Dnmt* and treated with 0.25 μg/mL doxycycline. Genomic DNA samples from HCT116 cells were used as both methylated and unmethylated *P16* controls (M &U Ctrl). (***A***) Bisulfite-DHPLC chromatograms indicate methylation changes in the 392-bp *P16* exon-1; (***B***) Quantitative RT-PCR results; (***C***) Western blot shows a gradual decrease in the concentration of P16 protein over time.


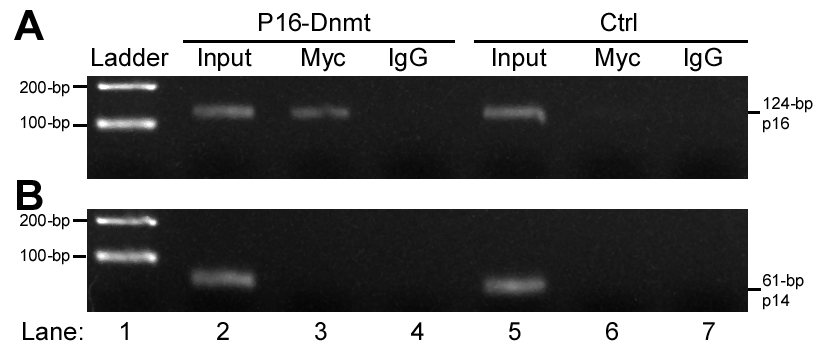


**Figure S2.** Chromatin immunoprecipitation (ChIP)–PCR analysis of P16-Dnmt- binding DNA fragments in BGC823 cells stably transfected with P16-Dnmt or empty control (Ctrl) vectors, and treated with 0.25 μg/mL doxycycline for 7 days. (***A***) ChIP-PCR analysis for *P16* DNA; (***B***) ChIP-PCR analysis for *P14* DNA

**
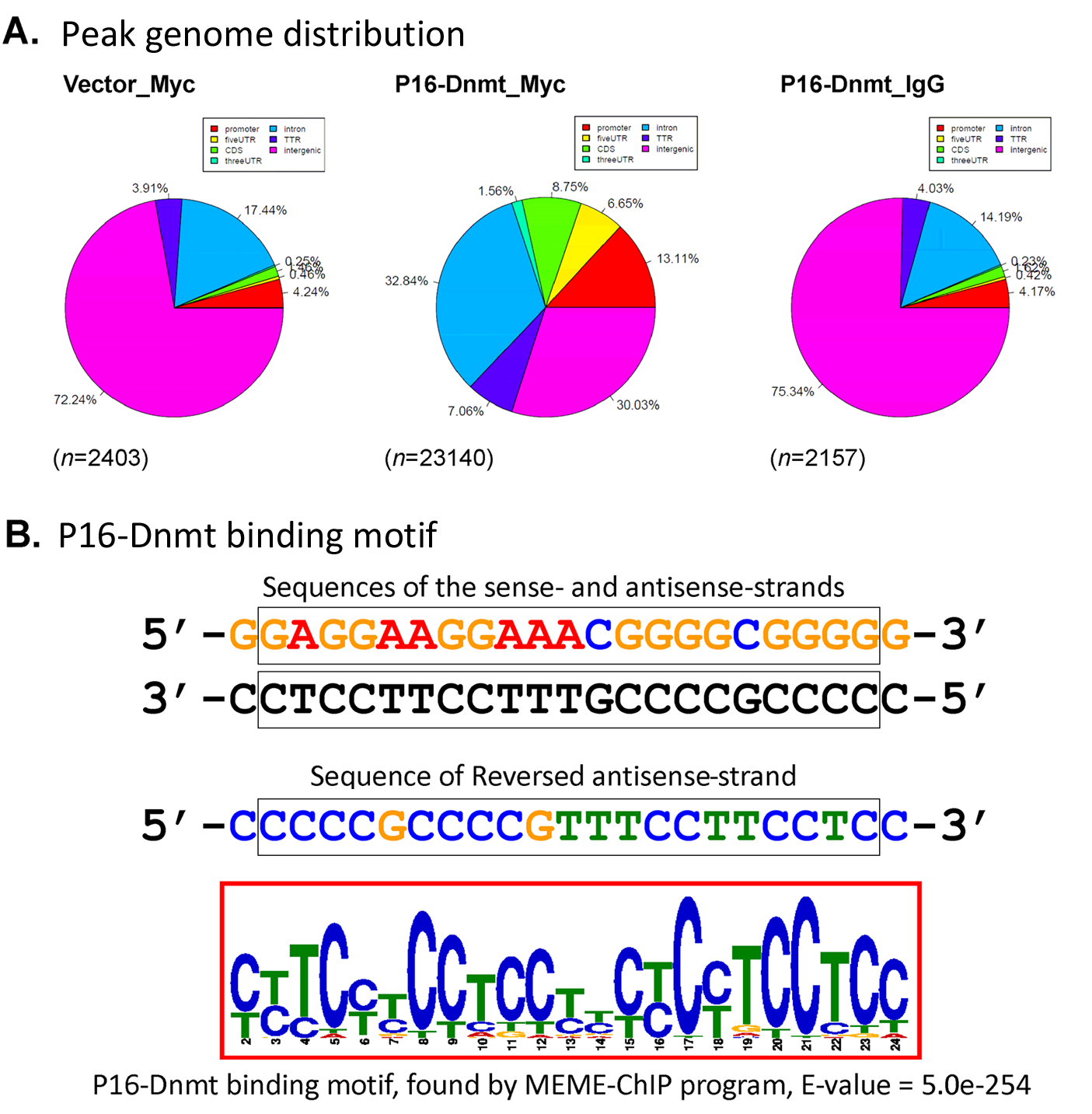
**

**Figure S3.** Characterization of chromatin-immunoprecipitation (ChIP)-sequencing results containing P16-Dnmt bound DNA fragments in BGC823 cells stably transfected with the *P16-dnmt* or control vector. (***A***) Genome distribution of P16-Dnmt binding DNA peaks. Number of total readout is also listed for each of the sequencing samples; Most of the binding DNA fragments are found in intergenic and intron regions in the genome; (***B***) Main P16-Dnmt binding motif (red rectangle) found using the MEME-ChIP program [39]. This sequence is highly consistent with the antisense-strand of the *P16* promoter used to design the *P16-*specific seven-zinc finger proteins [17]

**
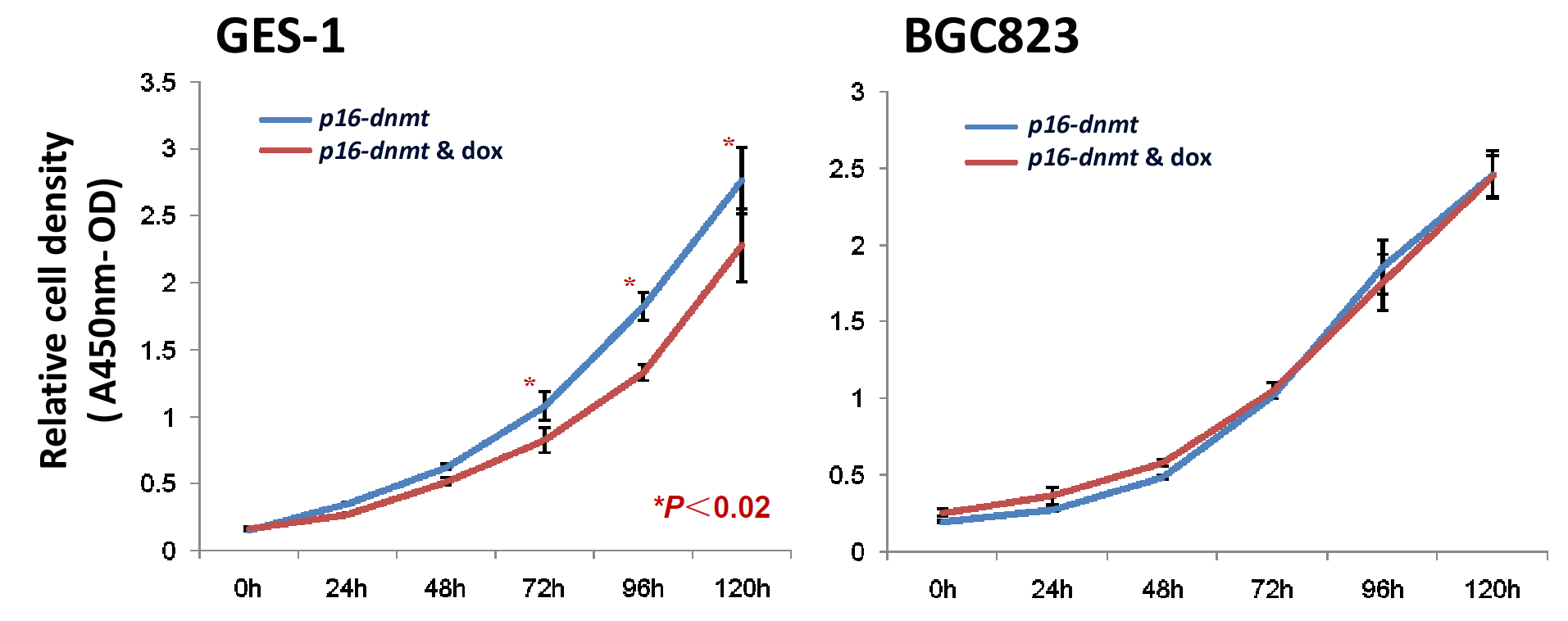
**

**Figure S4.** Cell proliferation analysis of GES-1 and BGC823 cell lines stably transfected with the *P16-dnmt* pTRIPZ vector. Each point represents the number of cells found in a single well averaged over 6 wells.


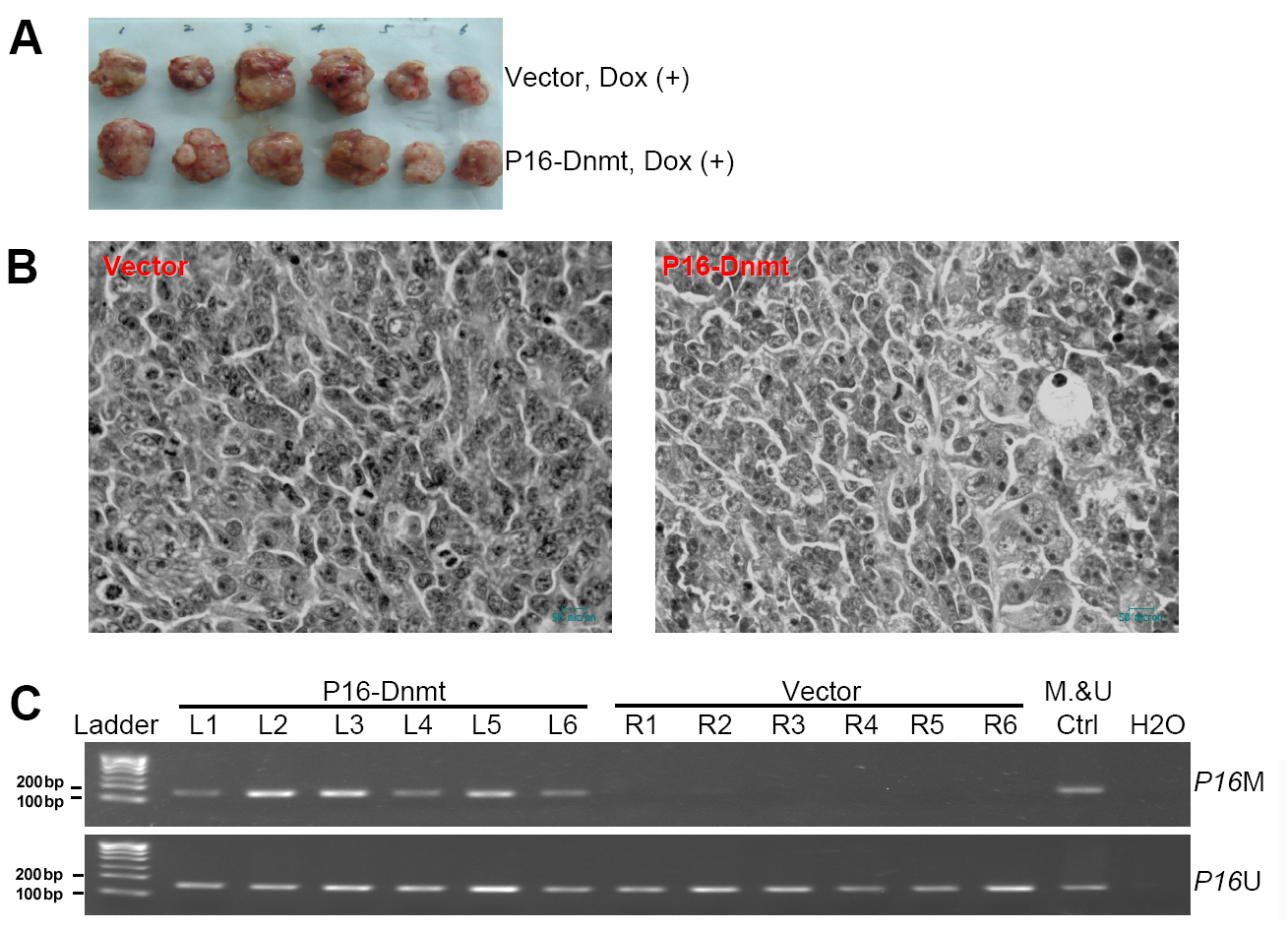


**Figure S5.** Analysis of xenografts taken from NOD SCID mice 28-days post injection of GES-1 cells stably transfected with P16-Dnmt or the pTRIPZ empty vector. (***A***) Xenografts taken from the mice show no difference in tumor burden. (***B***) Microscopy image of xenografts tissues (H.&E. staining). (***C***) Methylation-specific PCR detection of methylated- and unmethylated-*P16* alleles in these xenografts.

**
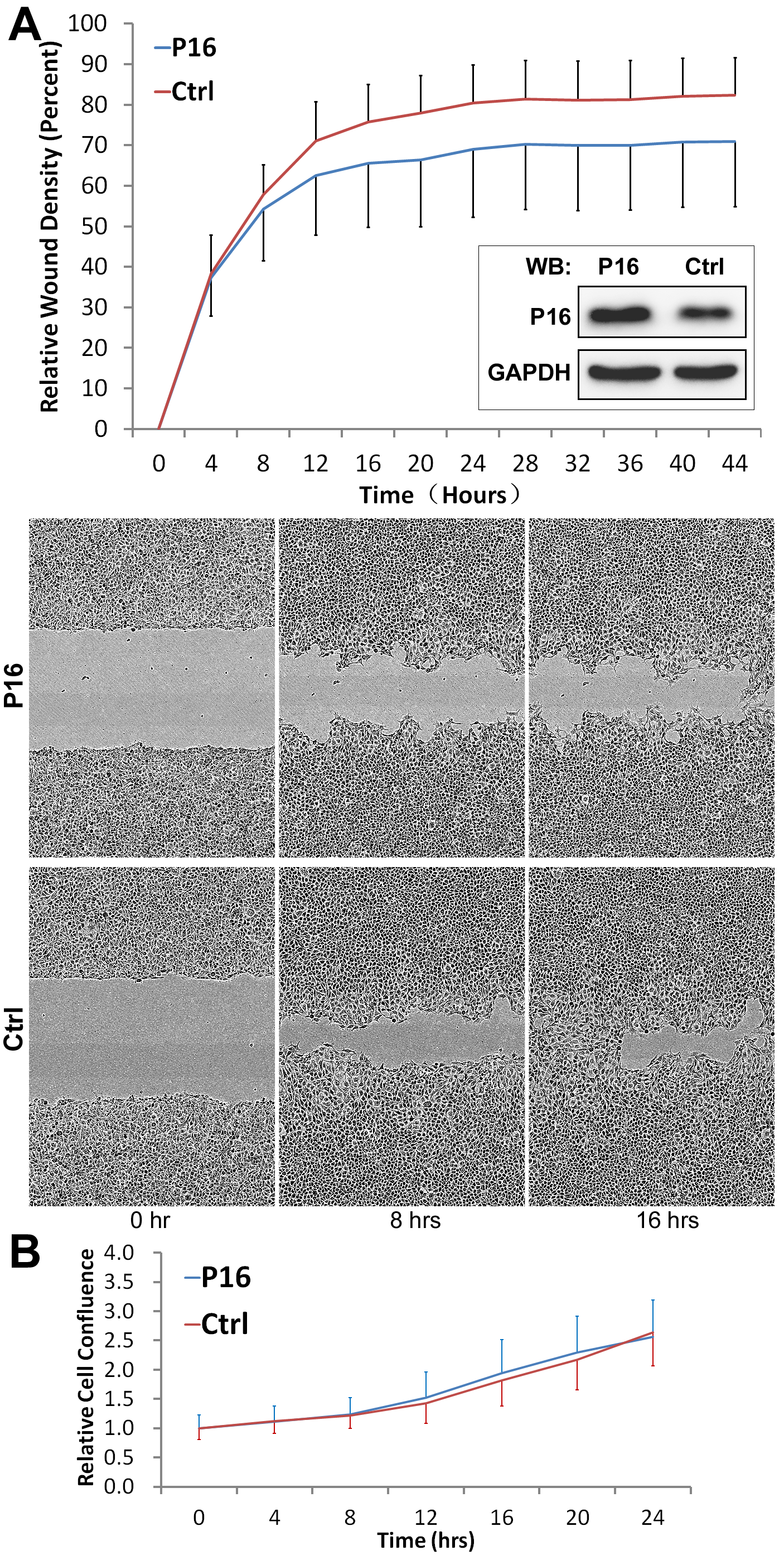
**

**Figure S6**. Effect of *P16* overexpression on migration and proliferation of HONE-1 cells. (***A***) Long-term, live-cell image analysis of the wound-healing assay comparing the migration of cells with enforced *P16* overexpression captured using the IncuCyte Zoom Cell Player. Western blot analysis is also displayed; (***B***) Proliferation curves generated using the long-term, live-cell image analysis of cells with enforced *P16* overexpression.
